# Isolation and enzyme bioprospection of bacteria associated to *Bruguiera cylindrica*, a mangrove plant of North Sumatra, Indonesia

**DOI:** 10.1016/j.btre.2021.e00617

**Published:** 2021-04-20

**Authors:** Jendri Mamangkey, Dwi Suryanto, Erman Munir, Apon Zaenal Mustopa, Mada Triandala Sibero, Lucas William Mendes, Adrian Hartanto, Steven Taniwan, Maria Julissa Ek-Ramos, Arman Harahap, Amit Verma, Edy Trihatmoko, Wendry Setiyadi Putranto, Lukas Pardosi, La Ode Adi Parman Rudia

**Affiliations:** a Department of Biology, Faculty of Mathematics and Natural Sciences, Universitas Sumatera Utara, Medan, 20155, Indonesia; b Research Center for Biotechnology, Indonesian Institute of Science, Jl. Raya Bogor Km. 46, Cibinong, West Java, 16911, Indonesia; c Department of Marine Science, Faculty of Fisheries and Marine Science, Universitas Diponegoro, Jl. Prof. Soedarto S.H., Tembalang, Semarang, 50275, Central Java, Indonesia; d Natural Product Laboratory, Integrated Laboratory for Research and Services, Universitas Diponegoro, Jl. Prof. Soedarto S.H., Tembalang, Semarang, 50275, Central Java, Indonesia; e Cell and Molecular Biology Laboratory, Center for Nuclear Energy in Agriculture CENA, University of Sao Paulo USP, Piracicaba, Brazil; f Department of Agricultural Sciences, University of Helsinki, Helsinki, 00014, Finland; g Universidad Autónoma de Nuevo León, Facultad de Ciencias Biológicas, San Nicolás de los Garza, Nuevo León, Mexico; h Faculty of Teacher Training and Education, Universitas Labuhanbatu, Rantauprapat, Indonesia; i Department of Biochemistry, College of Basic Science and Humanities, SD Agricultural University, Gujarat, 385506, India; j Department of Geography, Universitas Negeri Semarang, Semarang, 50229, Indonesia; k Faculty of Animal Husbandry, Padjadjaran University, Jatinangor, 45363, Indonesia; l Biology Study Program, Faculty of Agriculture, Universitas Timor, Kefamenanu, 85613, Indonesia; m Faculty of Mathematics and Natural Sciences, Halu Oleo University, Jalan H.E.A. Mokodompit, Kampus Baru, Kampus Hijau Bumi Tridharma Anduonohu, Kendari, 93232, Indonesia

**Keywords:** *Bruguiera cylindrica*, Extracellular enzymes, Mangrove, Protease, *Vibrio alginolyticus*

## Abstract

Mangrove-associated bacteria are of industrial interest due to their diverse and versatile enzyme properties. This study investigates the culturable bacteria from a wide range of habitat in a *Bruguiera cylindrica* mangrove ecosystem in North Sumatra. Screening of extracellular hydrolytic enzymes showed multiple potential traits in amylase, cellulase, chitinase, phosphatase, protease, and urease production by bacterial isolates. Molecular identification based on 16S rDNA region of a potential strain, *Vibrio alginolyticus* Jme3−20 is then reported as a newly proteolytic agent. The strain also showed a stable growth under salinity (NaCl) stress with considerable phosphate solubilization activities. Protease activity was enhanced by optimizing the 0.5 % (w/v) sucrose and soy peptone in the fermentation medium. SDS-PAGE and zymogram analysis showed the presence of a 35-kDa MW protease. Hence, our study revealed important insights into the bacterial diversity and activity in mangrove ecosystems, evidencing the importance of microbial exploration in this ecosystem.

## Highlights

• *Bruguiera cylindrica* is a mangrove plant in North Sumatra with limited information on its microbial endophytes.
• An enzyme bioprospection study was conducted revealing *Vibrio alginolyticus* as a prominent proteolytic strain.
• *Vibrio alginolyticus* Jme3-20 produced a multitude of extracellular enzymes such as amylase, cellulase, chitinase, phosphatase, and urease.
• This is the first report on finding *Vibrio alginolyticus* from mangrove area of North Sumatra.

## 1 Introduction

Mangrove ecosystems are located in the intertidal zones, comprising a large portion of the coastline in the tropical and subtropical regions of the Earth [1]. Mangroves are one of the most productive ecosystems in the world and are characterized by high rates of organic matter recycling and nutrients turnover between terrestrial habitats and ocean [2]. They occupy an important place in coastal ecosystems as they provide shelters for the breeding of birds, fish, crustaceans, shellfish, reptiles, mammals, and macrozoobenthos [3], being also essential for the maintenance of sea level and protection of the coast [4,5]. Mangrove forests have distinct characteristics if compared to other ecosystems, being limited by spatio-temporal variations in abiotic factors, such as temperature, salinity, nutrition, eutrophication, and pollution [[6], [7], [8]]. These conditions make mangroves hotspots for microbial diversity, and these communities play essential roles in the ecosystem functioning and maintenance [1]. Bacteria and fungi account for 91 % of the total microbial biomass living in mangroves, while algae and protozoa contribute for only 7 % and 2 %, respectively [9]. Mangrove microbes play crucial roles in biogeochemical cycles and supply plants and animals with primary nutritional sources [1,10,11]. Interestingly, bacteria are the major contributors to carbon flux in mangrove sediments [10,12]. The presence of bacteria exposes the multifunctionality of biotic interactions to the stability of mangrove ecosystems, acting as consumers and the main producers. Bacteria, as producers, spontaneously degrade mangrove litter as nutritional sources of carbon, sulfur, nitrogen, and phosphorus assimilated by mangrove plants [13,14]. Also, the roots of mangrove plants may benefit as a microhabitat to acquire and absorb nutrients produced by rhizospheric bacteria [14]. Thus, mangrove ecosystems provide valuable habitat for other organisms supported by the presence of diverse groups of bacteria. Enzymes are essential for the survival of bacteria in the natural environment as well as in host [15]. As a result, the exploration of bacterial communities in mangrove environments offers a great opportunity to find novel enzymes and other metabolites [[16], [17], [18]]. Besides, mangrove ecosystems have been proved as a potential source for finding various bacterial species that produce enzymes, proteins, antibiotics, and have salt-tolerant genes, all of them with the potential to be utilized in the future. Bacteria are important for enzyme production due to their high production capabilities, involving low use costs, and are easy to be genetically modified. Current microbial enzyme application extends to diverse sectors, including food processing, detergents, textiles, agriculture, pharmacy, medical therapy, and molecular biology. Until now, the exploration of mangrove bacterial strains is still undergoing or in a developmental stage and has a great potential to be used in the biotechnological industry. Considering that mangrove ecosystems have been highlighted as critical environments needing urgent attention and conservation policies, the exploration of the bacterial diversity is of paramount importance for a better understanding of the ecosystem. Therefore, in this study, we screened hydrolase-producing bacterial isolates inhabiting the mangrove sediment and insects associated with the tree *Bruguiera cylindrica*. For this, we performed enzymatic profiling of α-amylase, β-amylase, protease, cellulase, chitinase, and phosphate solubilizers, as potential extracellular enzyme producers with a high prospect for applications in the future.

## 2 Materials and method

### 2.1 Sampling site

**Fig. 1:**
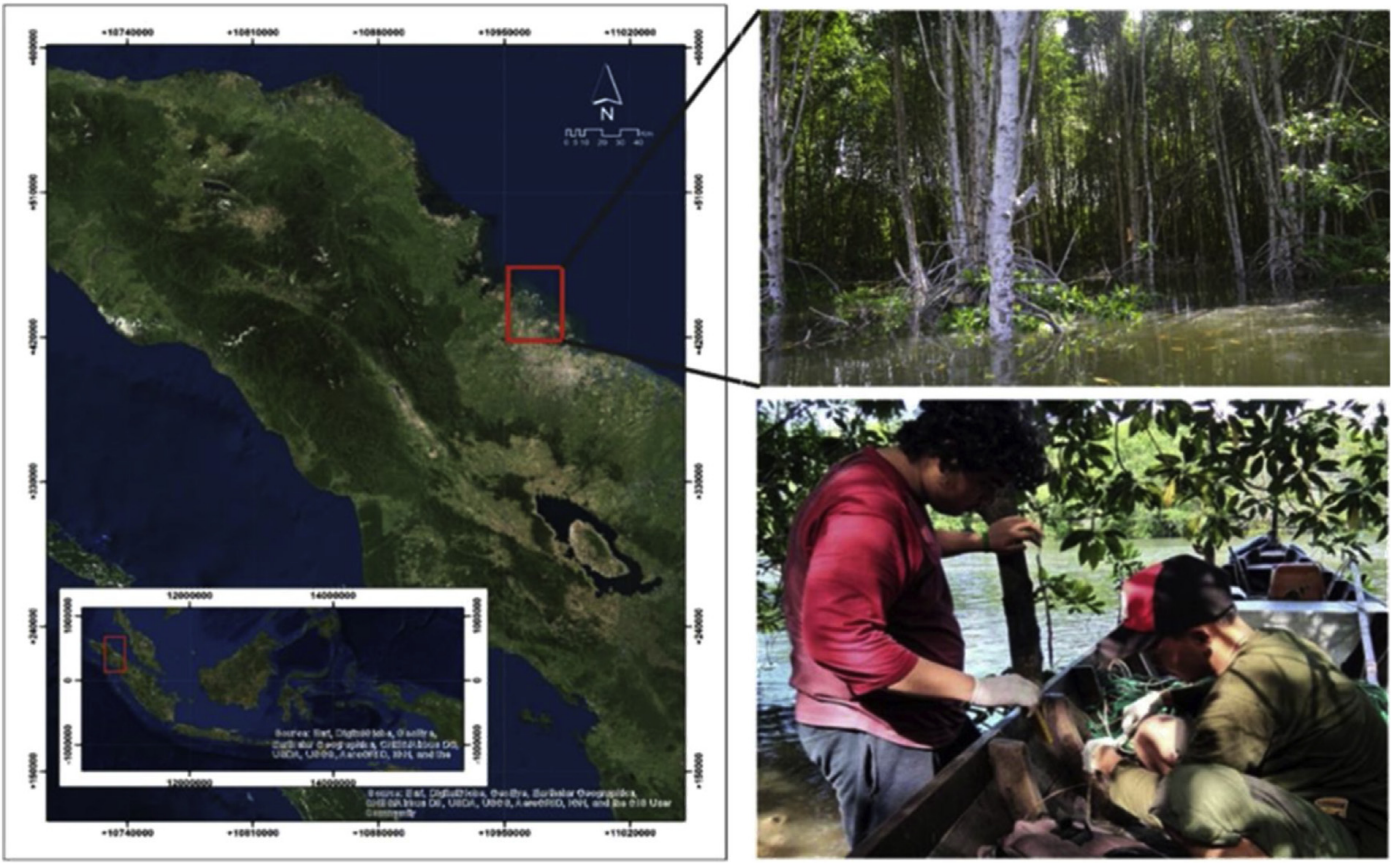
Sampling site map location. Samples were collected in Pantai Gading mangroves, located in Secanggang district, Langkat regency, North Sumatra, Indonesia.

Insects and mangrove sediments were collected from mangroves of Pantai Gading Secanggang (3˚50.189′ N, 98˚35.385′E) Langkat regency, North Sumatra, Indonesia (Fig. 1). Insects were collected from bark samples, while sediments were taken from a ± 30 cm- diameter area surrounding roots. Sediments were collected from different depths (10, 20, and 30 cm) using a 88 cm-diameter PVC pipe. Each sediment sample was placed into a 17 × 25 cm sterile zip-lock plastic and stored in cold conditions (4 °C) until laboratory experimentation.

### 2.2 Isolation of mangrove-associated bacteria

The isolation of bacteria was performed according to Mamangkey et al. [19]. Briefly, insect samples were grinded to powder by using mortar and pestle and bacteria was extracted from 1 g in 9 mL NaCl (0.9 % w/v) with a serial dilution of 10−1 to 10−9. A 100 μl of sample suspension from three dilutions (10^−7^, 10^−8^, 10^−9^) was poured and spread on nutrient agar plate supplemented with 1.8 % NaCl and incubated at 37 °C for 24 h. Distinct morphologies among isolated bacteria were designated as different isolates and used in enzymatic assays as described below.

### 2.3 Qualitative screening of hydrolytic enzymes

#### 2.3.1 Amylase

The screening of α-/β-amylase-producing bacteria was done by using a starch medium with a composition of (w/v)/L: starch (10 g for α-amylase and 5 g for β-amylase), yeast extract (1 g), NaCl (18 g), agar (15 g), dissolved in distilled water. Bacterial isolates were spotted on top of the agar medium and incubated at 30 °C for 21, 24, and 27 h to observe hydrolysis rates [20,21]. After incubation, a lugol’s iodine solution (1 g Iodine in 2% KI solution) was poured into agar medium and settled for 10 min, until clear zones were formed around bacterial colonies [22]. The blue color indicated negative hydrolysis of α-/β-amylases while clear zones indicated positive hydrolysis.

#### 2.3.2 Protease

The screening of proteolytic bacteria was conducted by using skim milk medium with a composition of (w/v) per litre: peptone (4 g), yeast extract (1 g), skim milk (12 g), NaCl (18 g), agar (15 g), dissolved in distilled water [20]. Bacterial isolates were spotted on top of the agar medium and incubated at 30 °C for 21, 24, and 27 h to observe hydrolysis rates. Clear zones around bacterial colonies indicated proteolysis positive results.

#### 2.3.3 Cellulase

The screening of cellulolytic bacteria was performed by using CMC-Bushnell Haas medium with a composition of (w/v) per litre: CMC (10 g), K_2_HPO_4_ (1 g), KH_2_PO_4_ (1 g), MgSO_4_⋅7H_2_O (0.2 g), NH_4_NO_3_ (1 g), FeCl_3_⋅6H_2_O (0.05 g), NaCl (18 g), CaCl_2_ (0.02 g), Agar (15 g), dissolved in distilled water [23]. Bacterial isolates were spotted on top of the agar medium and incubated at 30°C for 96 h to allow maximum growth. Clear zones around bacterial colonies indicated a proteolysis positive result. After incubation, the congo red solution (0.3 %) was poured into the agar medium and settled for 20 min at 25 °C. The agar medium was washed using NaCl 1 M to clarify the clear zones formed around colonies, which indicated cellulolysis positive results.

#### 2.3.4 Chitinase

The screening of chitinase-producing bacteria was done by using colloidal chitin medium with composition of (w/v) per litre: Na_2_HPO_4_ (6 g), KH_2_PO_4_ (3 g), NH_4_Cl (1 g), NaCl (0.5 g), yeast extract (0.05 g), agar (15 g), colloidal chitin 1% (w/v) dissolved in distilled water. Preparation of colloidal chitin was based on Saima et al. [24]. Bacterial isolates were spotted on top of the agar medium and incubated at 30 °C for 96 h to allow maximum growth. Clear zones around bacterial colonies indicated chitinolysis positive results.

#### 2.3.5 Phosphatase

The screening of phosphatase-producing bacteria was done by using Sperber medium plate with slight modification containing insoluble phosphate. Sperber medium was composed of 10 g glucose, 0.5 g yeast extract, 0.2 g KCl_2_, 0.3 g MgSO_4_.7H_2_O and supplemented with 5 g Ca_3_(PO_4_)_2_ or tricalcium phosphate (TCP) and 15 g agar (in solid medium) at pH 7.4, dissolved in distilled water (1 L). To examine phosphate solubilization capabilities, 5 μL (∼10^8^ CFU/mL) was streaked in a zig-zag pattern on top of Sperber medium plate and incubated at 25 ± 2 °C for 48, 72, 120, 144, and 168 h to observe the hydrolysis rates. Clear zones around bacterial colonies indicate a positive result of soluble phosphates released from insoluble phosphates.

#### 2.3.6 Urease

The screening of urease-producing bacteria was done by using a modified urea medium with a composition of (w/v) per litre: urea (20 g), NaCl (18 g), KH_2_PO_4_ (2 g), peptone (1 g), dextrose (1 g), phenol red (0.012 g), agar (15 g) dissolved in distilled water [25]. Bacterial isolates were stabbed on top of the agar medium and incubated at 30 °C for 72 h. The color change of medium to pink indicated ureolysis positive results.

### 2.4 Salinity tolerance and P solubilization assay

Salinity tolerance of the strain Jme3−20 was assessed by measuring changes in the optical density of culture grown in Sperber’s Broth (SB) medium, supplemented with NaCl at the following concentrations: 0, 2, 4, 6, 8, and 10 % at 0.5 M. Bacterial growth was monitored using spectrophotometer (600 nm) at 144 h (optimal time of phosphatase production) after inoculation. Then, the phosphatase activity was determined based on the concentration of dissolved phosphate. Assay for quantifying phosphate solubilization, the pure bacterial colonies were grown in a liquid Sperber medium at 26 ± 2 °C and 120 rpm for 144 h. Thereafter, the suspensions were centrifuged at 10,000 rpm for 10 min at 4 °C in order to separate the solubilized phosphate from bacterial cells, suspended particles, and insoluble phosphate. The soluble phosphate in the supernatant was quantified by the vanadate- molybdate method [26]. To concentrate the soluble phosphate, a calibration with KH_2_PO_4_ was performed. The concentration of soluble phosphate was measured in triplicates and absorbance at 420 nm using UV Mini-1240 spectrophotometer (Shimadzu Co., Japan). One unit of activity was defined as the amount of enzyme that releases 1 μmol of inorganic phosphate during 1 min under the assay conditions.

### 2.5 Molecular identification of potential strain

The promising bacterial strain with a wide range of enzyme activities, namely Jme3−20, was identified according to Sibero et al. [27]. For this, the primers 27 F (5′-AGA GTT TGA TCC TGG CTC AG-3′) and 1492R (5′- GGT TAC CTT GTT ACG ACT T-3′) were used for bacterial 16S rDNA PCR amplification. PCR products were sequenced by 1^st^ Base Laboratories Sdn Bhd, Malaysia, and Basic Local Alignment Search Tool (BLAST) was carried out to identify closer related sequences of known bacteria. The phylogenetic tree was reconstructed using the MEGA X software package with the neighbor-joining method with 1000 number of bootstrap replications [28].

### 2.6 Optimization of extracellular protease

Protease activity produced by Jme3−20 was assayed progressively based on its preliminary screening results. Optimization of extracellular protease production was done by using an enriched medium with the following composition (w/v) in 1 L: each one of these N-sources (peptone, keratin, skim milk, yeast extract, beef extract, soy peptone) (10 g), glucose (5 g), MgCl_2_.6H_2_O (4 g), KCl (1 g) dissolved in distilled water.

### 2.7 Protease assay and protein quantification

Extracellular protease activity was observed at each 24 h, during 168 h. Protease activity was assayed according to Enyard [29], with slight modifications. Four solutions were prepared (w/v) in 1 L, as follows: Solution A containing Na_2_CO_3_ (0.2 g) in 10 mL NaOH 0.1 N; Solution B containing CuSO_4_.5H_2_O (0.05 g) in 10 mL Na_2_C_4_H_4_O_6_; Solution C was made by mixing solution A and B with a ratio of 5:1 (v/v); Solution D was made by mixing Folin- Ciocalteu and double distilled water with a ratio of 1:1 (v/v) [30]. Protease assay was performed in 96-well plates. Six μL of the crude enzyme was added to a 6 μL mixture of 25 mM Tris−HCl buffer pH 7.4 and casein (1%, w/v) and double distilled water was used as blank. Tyrosine was used for amino acid estimation with the range of 50, 100, 150, 250, 300, 350, and 400 (μL/mL). The mixture of enzyme and substrate was incubated at 37 °C for 30 min. The enzymatic reaction was stopped by adding 12 μL TCA solution, 143 μL solution C, and 30 μL solution D. Reaction mixture was centrifuged and measured in a spectrophotometer at 540 nm absorbance. Protein or enzyme quantification was assayed using Bicinchoninic acid (BCA) kit protein (Thermo Scientific™). 10 μL of crude enzyme solutions were mixed with 200 μL of working reagent in 96-well plates. The working reagent was prepared by mixing solutions A and B with a ratio of 50:1. Samples and working reagent were put into plates at a ratio of 1:20. The plates were then incubated at 37 °C for 30 min. The absorbance was measured at 540 nm using an ELISA reader. Bovine serum albumin was used as protein standard at a range of 2000, 1500, 1000, 750, 500, 250, 125, and 25 (μL/mL), as suggested by the kit manual.

### 2.8 Determination of bacterial growth

The bacterial growth during optimization was measured in a spectrophotometer U-3900H (Hitachi) at OD_600_. Cells were measured at each 24 h during 168 h, to match the protein content measurements and protease assay.

### 2.9 Gel filtration chromatography

The process of purifying Jme3−20 proteins was conducted by using Sephadex G-50 gel column chromatography. The supernatants obtained from bacterial cultures were filtered through 0.45 μm PVDF syringe filters (Millipore, USA). Then a pellet (resin binding) was obtained by precipitation in ammonium sulfate 50 % and dialyzed by using a 10 kDa cut-off membrane and adding 5 mL buffer A (25 mM Tris−HCl, pH 8) to dissolve the pellet. The dialysis apparatus was submerged into 600 mL buffer B (25 mM Tris−HCl, pH 7.4) and gently stirred for 24 h at 5 °C. Buffer B was changed every 8 h. Furthermore, dialyzed protein extract was purified using a Sephadex G-50 (Sigma-Aldrich) gel filtration chromatography column (fractionation range 1.5–30 kDa). The column was prepared using 25 mM Tris−HCl, pH 8.0. Protein extract was gently poured onto the column and eluted with 25 mM Tris−HCl, pH 8.0. The fractions obtained were collected into 1.5 mL microcentrifuge tubes and protein concentration was determined by using a UV–vis spectrophotometer at 280 nm. The fraction with the highest protein concentration was used for further protein molecular weight determination.

### 2.10 SDS-PAGE and zymography

SDS-PAGE analysis was performed in 10 % (w/v) polyacrylamide resolving gel and 5% (w/v) polyacrylamide stacking gel. Samples and loading dye with a total volume of 15 μl were incubated at 100 °C for 5 min to denature proteins. The denatured samples were run at 110 V for 90 min. After the protein separation was complete, the gel was removed and Coomassie® brilliant blue R-250 stained and further placed in methanol acetate solution (250 mL distilled water, 200 mL methanol, and 50 mL glacial acetic acid). Zymography was conducted to predict in situ protein hydrolysis (casein) yet confirming its enzymatic properties. Zymogram obtained by running samples into a 10 % separating gel added with 0.2 % soy peptone. After the separation process, the gel was incubated in 2.5 % Triton® X-100 at 37 °C for 1 h. The gel was incubated in 50 mM Tris−HCl buffer (pH 8) overnight then stained in 0.05 % Coomassie brilliant blue solution for 2 h. Destaining solution [acetic acid: methanol: distilled water (10:20:70 v/v)] was used until white bands appeared.

## 3 Results

### 3.1 Sampling sites

The mean temperature during sampling in Pantai Gading mangrove was ranged from 26 to 27 °C and pH 6.7–6.8 in the five sampling sites.

### 3.2 Isolation of bacteria associated with *Bruguiera cylindrica*

**Fig. 2:**
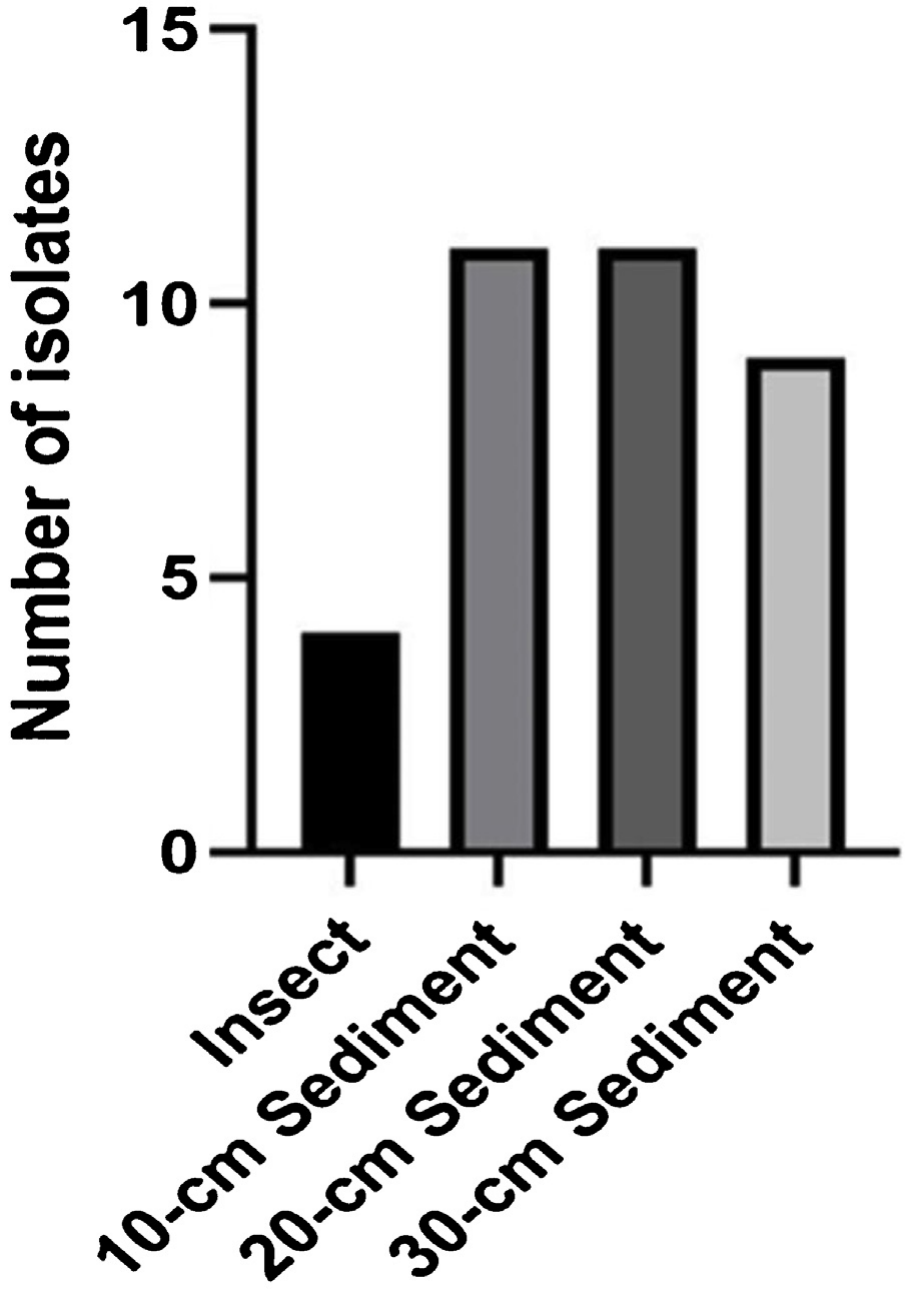
Number of bacterial isolates obtained from different sampling types. Insect (4 isolates), sediment 10 cm (11 isolates), 20 cm (11 isolates) and 30 cm (9 isolates).

Bacteria were isolated from five different sampling sites around the Pantai Gading mangrove ecosystem of the Secanggang district. The samples taken were insects and mangrove sediments at three different depths (10, 20 and 30 cm depth). The total number of isolated bacteria was 35 (Fig. 2). The number of bacterial isolates obtained per sediment varied according to the depth. 10−20 cm depth samples harbored a larger number of bacteria than 30 cm samples. The presence of bacteria in the sediment was strongly influenced by the content of the elements utilized as nutrients for bacterial growth. The results of the decomposition of the mangrove litters, i.e. stem, leaves, and propagules of mangroves were mostly accumulated on the top of the soil: the decomposed mangrove litters then cause more availability of nutrients such as carbon, nitrogen, phosphorus, magnesium, calcium, and other elements on the soil surface. These results are supported by Mendes and Tsai [31] study that showed P, K, Ca, and Mg the highest physicochemical content in mangrove sediments at 10 cm depth.

### 3.3 Screening of amylolytic bacteria

**Fig. 3:**
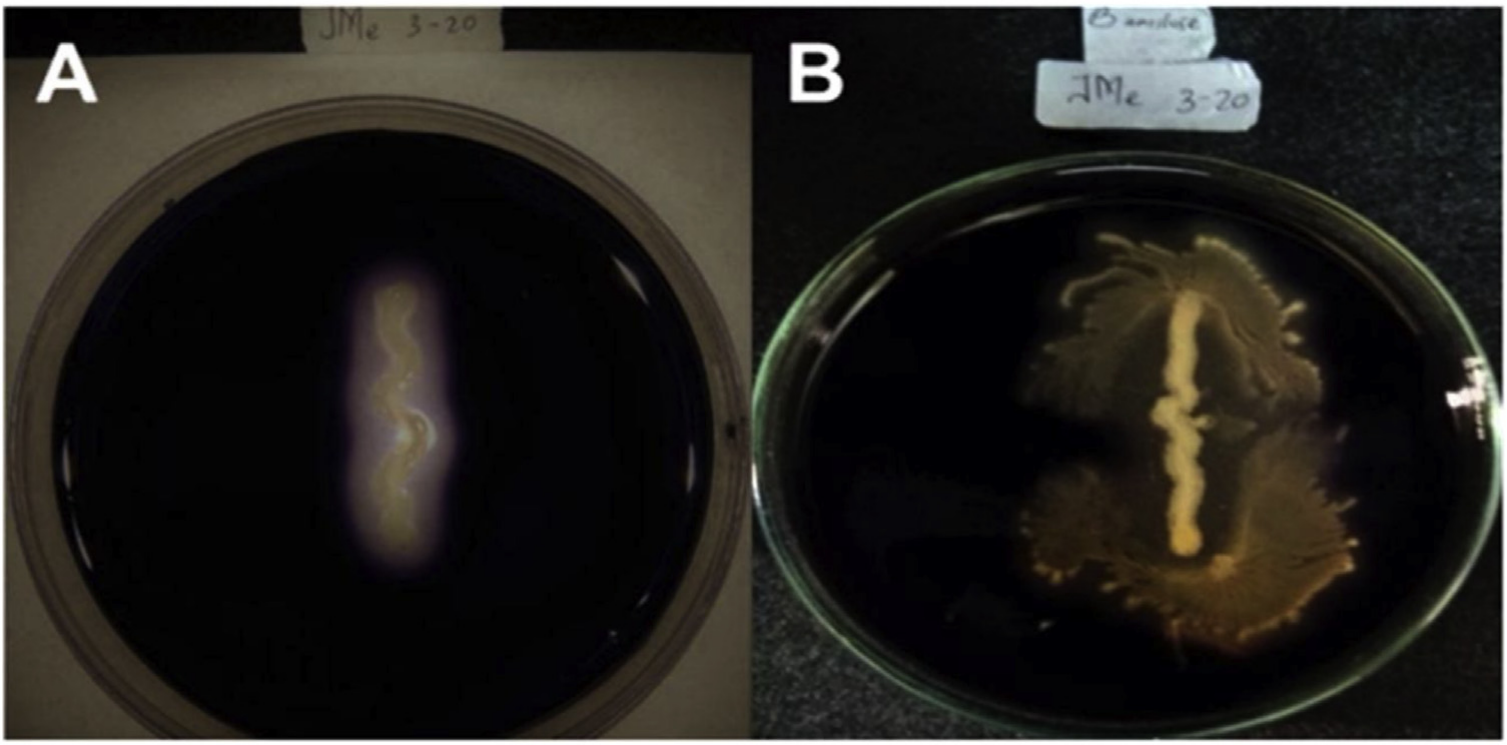
Representative positive results of isolate Jme 3-20, showing clear zones surrounding colonies (unstained), indicating starch hydrolysis.(A) α-amylase assay, (B) β-amylase assay.

In general, most of the bacterial isolates were able to synthesize extracellular α-/β-amylases (Table 1). 18 out of 35 isolates showed the highest production of α-amylase within 27 h, while 17 showed their highest rate at 24 h. Besides, four isolates were observed to produce the highest β-amylase within 27 h and the remaining 31 showed their highest production at 24 h. The greater number of β-amylase-producing bacteria that could produce the enzyme in a shorter time was probably due to faster hydrolysis, which was shown by clear zones formed around their colonies (Fig. 3).

**Table 1:** α-/β-amylases activities of bacteria associated with *Bruguiera cylindrica*.

Isolate Code	α-amylase			β-amylase		
	Observation (hr)					
	21	24	27	21	24	27
JMb 2−20	+	+	++	+	++	++
JMa 2−20	+	+	+	+	+	+
JMb 3−20	+	++	++	+	++	++
JMc 3−20	+	++	++^R^	+	++	++^R^
JMb 1−10	+	+	++	+	++	++
JMd 3−20	+	++	++	+	++	++
JMc 1−30	+	++	+++ ^R^	++	+++	+++ ^R^
JMc 2−30	+	++	++	+	++	+++
JMd 1−10	+	++	+++	++	+++	+++
Jme 1−10	+	++	+++	++	+++	+++
JMa 1−30	+	++	++	+	++	++
JMb 3−30	+	++	+	+	++	++
JMb 1−20	+	++	++	++	++	++
JMb 1−30	+	++	++ ^R^	++	++	++ ^R^
JMc 1−20	+	++	+++	++	++	+++
JMb 2−30	+	++	+++	++	++	+++
JMa 2−10	+	+	++	++	++	++
JMa 2−30	+	+	++	++	++	++
JMf 1−10	+	++	+++	++	++	+++
JMa 3−30	+	+	++	++	++	++
JMb 3−10	+	++	+++ ^R^	++	+++	+++ ^R^
JMa 3−10	+	++	+++	++	+++	+++
JMc 2−10	+	++	+++	++	+++	+++
JMc 3−30	+	++	++ ^R^	++	+++	++ ^R^
JMb 2−10	+	++	++ ^R^	+	++	++ ^R^
JMc 1−10	+	++	++	++	+++	++
JMa 3−20	+	++	++ ^R^	++	++	++ ^R^
JMf 3−20	+	++	++	+	++	++
Jme 3−20	+	++	++ ^R^	++	+++	+++ ^R^
JMa 1−10	+	+	+	+	+	+
JMa 1−20	+	++	++	++	++	++
ISSC2 a	+	+	++	+	++	++
ISSC2 b	+	+	++	+	++	++
ISSC1 a	+	+	++	+	++	++
ISSC1 b	+	+	++	++	++	++

^R^: Colonies with rhizoid appearance.

+ low, ++ medium, +++ high activity.

### 3.4 Screening of proteolytic bacteria

**Fig. 4:**
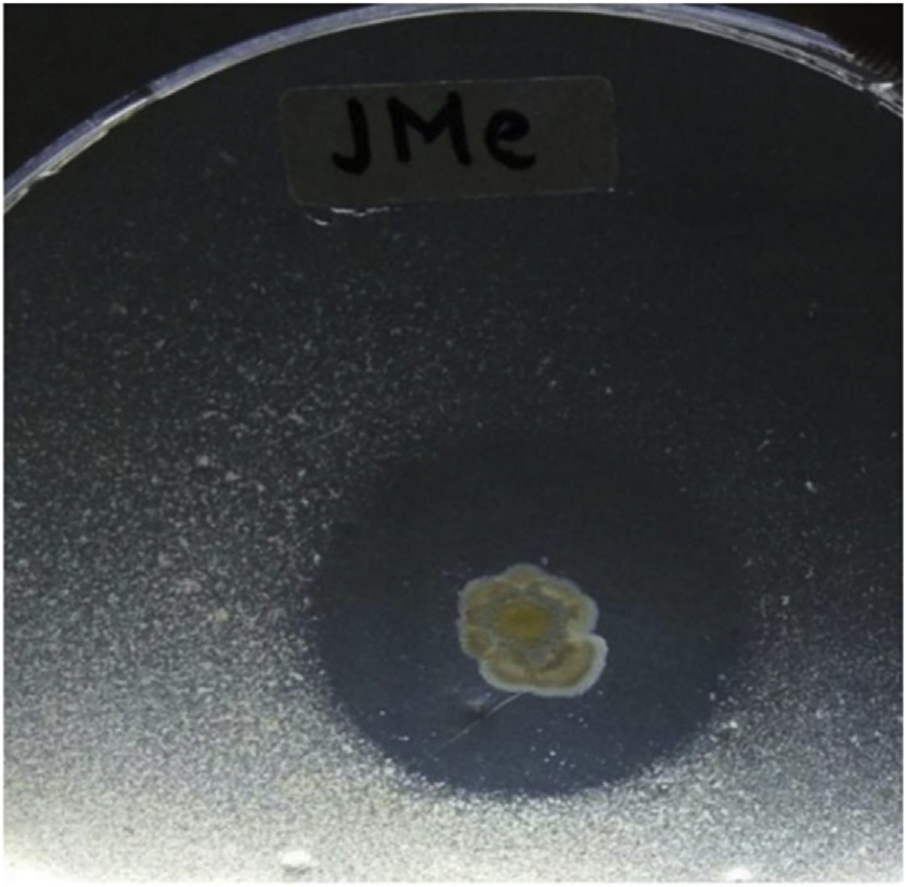
Bacterial isolate Jme3-20 positive results showing clear zones around colonies, indicating hydrolysis of skim milk.

Among 33 protease-producing isolates obtained in this study, only two of them showed the highest activity after 27 h incubation (Table 2). Two isolates, from the original 35, did not show any proteolytic activity while one isolate had very low proteolytic activity. Clear zones formed around colonies indicated positive results on skim milk hydrolysis, which was used as a carbon source in the medium (Fig. 4).

**Table 2:** Protease activity of bacteria associated with *Bruguiera cylindrica*.

Isolate Code	Observation (hr)		
	21	24	27
JMb 2−20	+	+	+
JMa 2−20	–	–	–
JMb 3−20	+	+	+
JMc 3−20	+^a^	+	+
JMb 1−10	+^a^	+	+
JMd 3−20	+^a^	+	+
JMc 1−30	+	+	+
JMc 2−30	+	+	+
JMd 1−10	+	+	+
Jme 1−10	+	+	+
JMa 1−30	+	+	+
JMb 3−30	+	+	+
JMb 1−20	+	+	+
JMb 1−30	+	+	+
JMc 1−20	+	+	+
JMb 2−30	+	+	+
JMa 2−10	+	+	+
JMa 2−30	+	+	+
JMf 1−10	+	+	+
JMa 3−30	++	+++	+++
JMb 3−10	+	+	+
JMa 3−10	+	+	+
JMc 2−10	+	+	+
JMc 3−30	+	+	+
JMb 2−10	+	+	+
JMc 1−10	+	+	+
JMa 3−20	+	+	+
JMf 3−20	+	+	+
Jme 3−20	+ +	+ ++	+ ++
JMa 1−10	+	+	+
JMa 1−20	+	+	+
ISSC2 a	+^a^	+^a^	+^a^
ISSC2 b	+	+	+
ISSC1 a	–	–	–
ISSC1 b	+	+	+

+^a^: colony grow with a very low protease activity.

- none, + low, ++ medium, +++ high activity.

### 3.5 Screening of cellulolytic bacteria

**Fig. 5:**
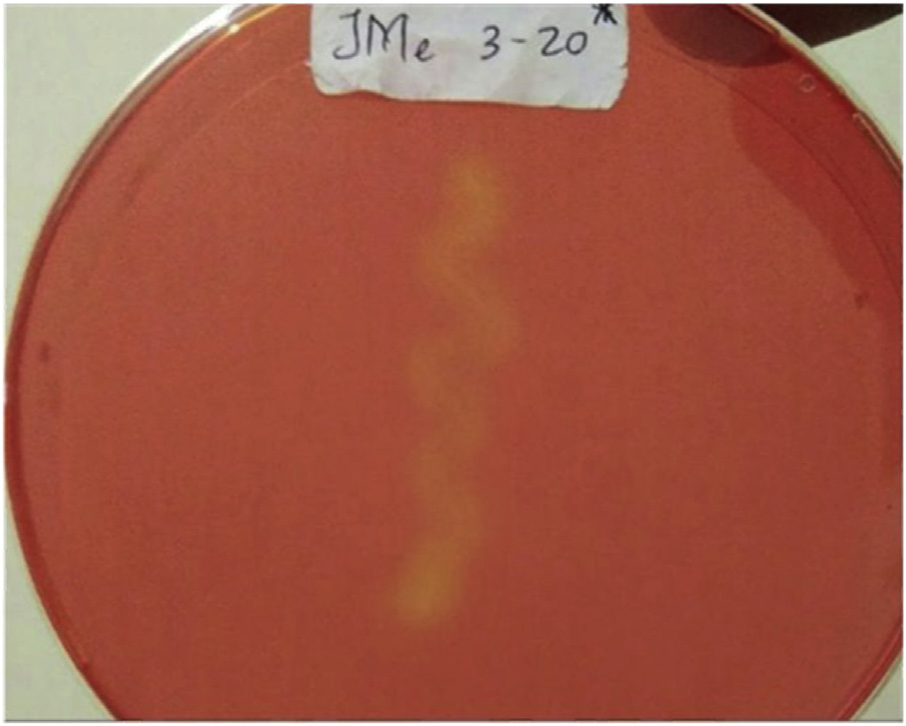
Jme3-20 positive results showing clear zones around colonies indicating hydrolysis of Carboxyl Methyl Cellulose (CMC).

Only seven isolates were observed as cellulase-producing strains in this study (Table 3). Besides, the duration of cellulose hydrolysis was longer than in previous screenings, which required a 96-h incubation to exhibit positive results. Clear zones formed around colonies indicated positive results of CMC hydrolysis. CMC was used as a carbon source in the medium (Fig. 5).

**Table 3:** Cellulase activity of bacteria associated with *Bruguiera cylindrica*.

Isolate Code	Incubation (hr)
	96
JMb 2−20	–
JMa 2−20	–
JMb 3−20	+
JMc 3−20	–
JMb 1−10	–
JMd 3−20	–
JMc 1−30	–
JMc 2−30	–
JMd 1−10	–
Jme 1−10	–
JMa 1−30	–
JMb 3−30	–
JMb 1−20	–
JMb 1−30	–
JMc 1−20	–
JMb 2−30	–
JMa 2−10	–
JMa 2−30	–
JMf 1−10	–
JMa 3−30	–
JMb 3−10	–
JMa 3−10	–
JMc 2−10	–
JMc 3−30	–
JMb 2−10	–
JMc 1−10	–
JMa 3−20	+
JMf 3−20	–
Jme 3−20	+
JMa 1−10	–
JMa 1−20	–
ISSC2 a	+
ISSC2 b	+
ISSC1 a	+
ISSC1 b	+

- none, + low cellulase activity.

### 3.6 Screening of chitinolytic bacteria

**Fig. 6:**
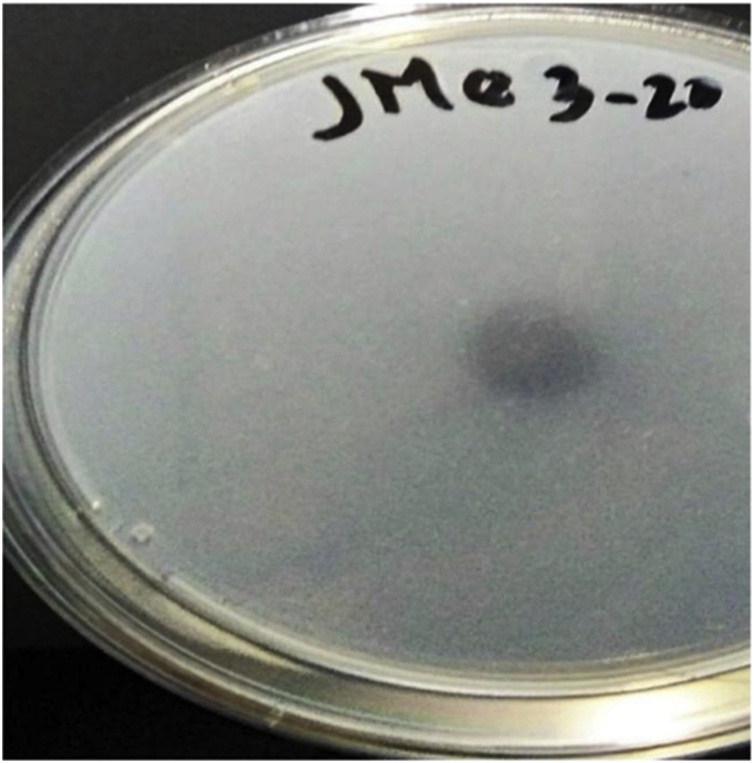
Jme3-20 positive results showing the clear zones around colonies indicating hydrolysis of colloidal chitin.

In this study only one isolate with chitinase activity was obtained, namely Jme 3−20. The chitinolytic activity was observed after 6 days of incubation in colloidal chitin agar (Fig. 6).

### 3.7 Screening of phosphate solubilizing bacteria

**Fig. 7:**
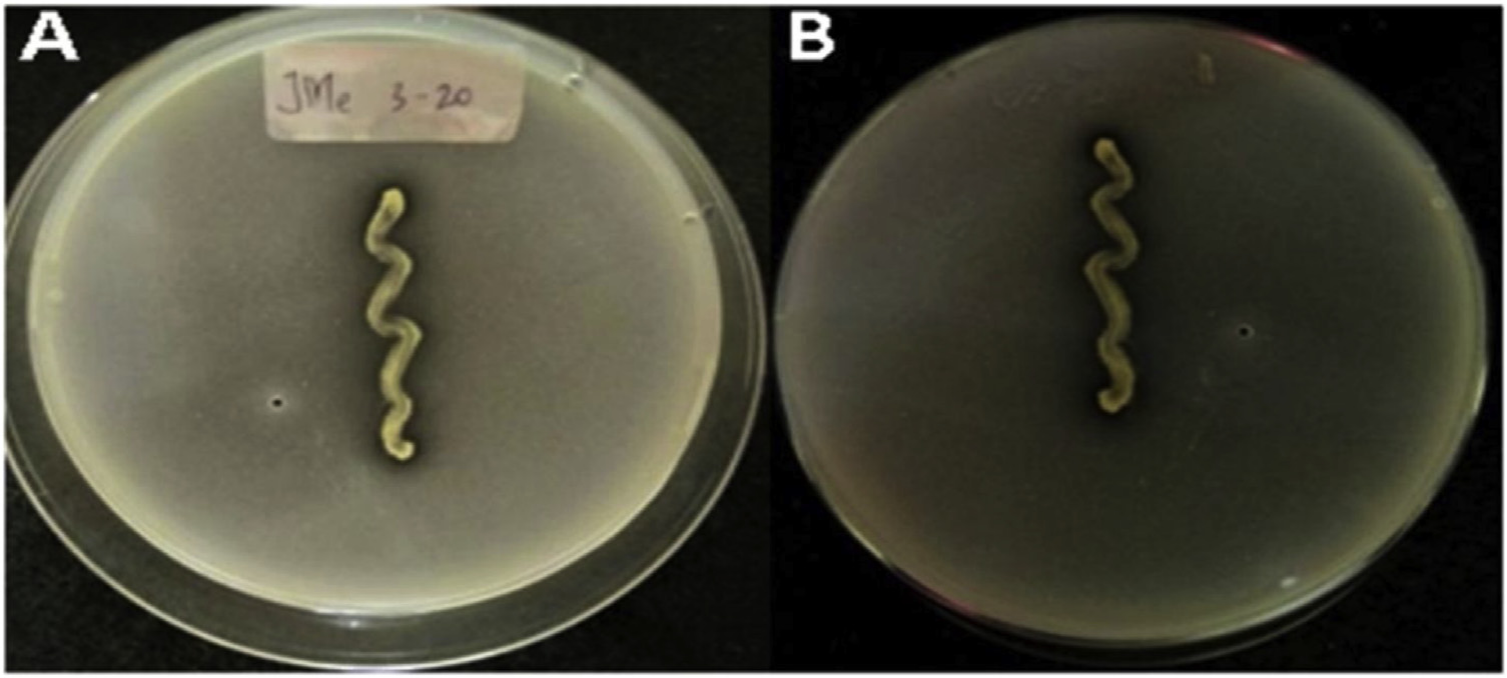
Jme3-20 positive results showing clear zones around colonies, indicating solubilization of Ca_3_(PO_4_)_2_ after 144 h of incubation. (A) Surface, (B) Reverse.

**Fig. 8:**
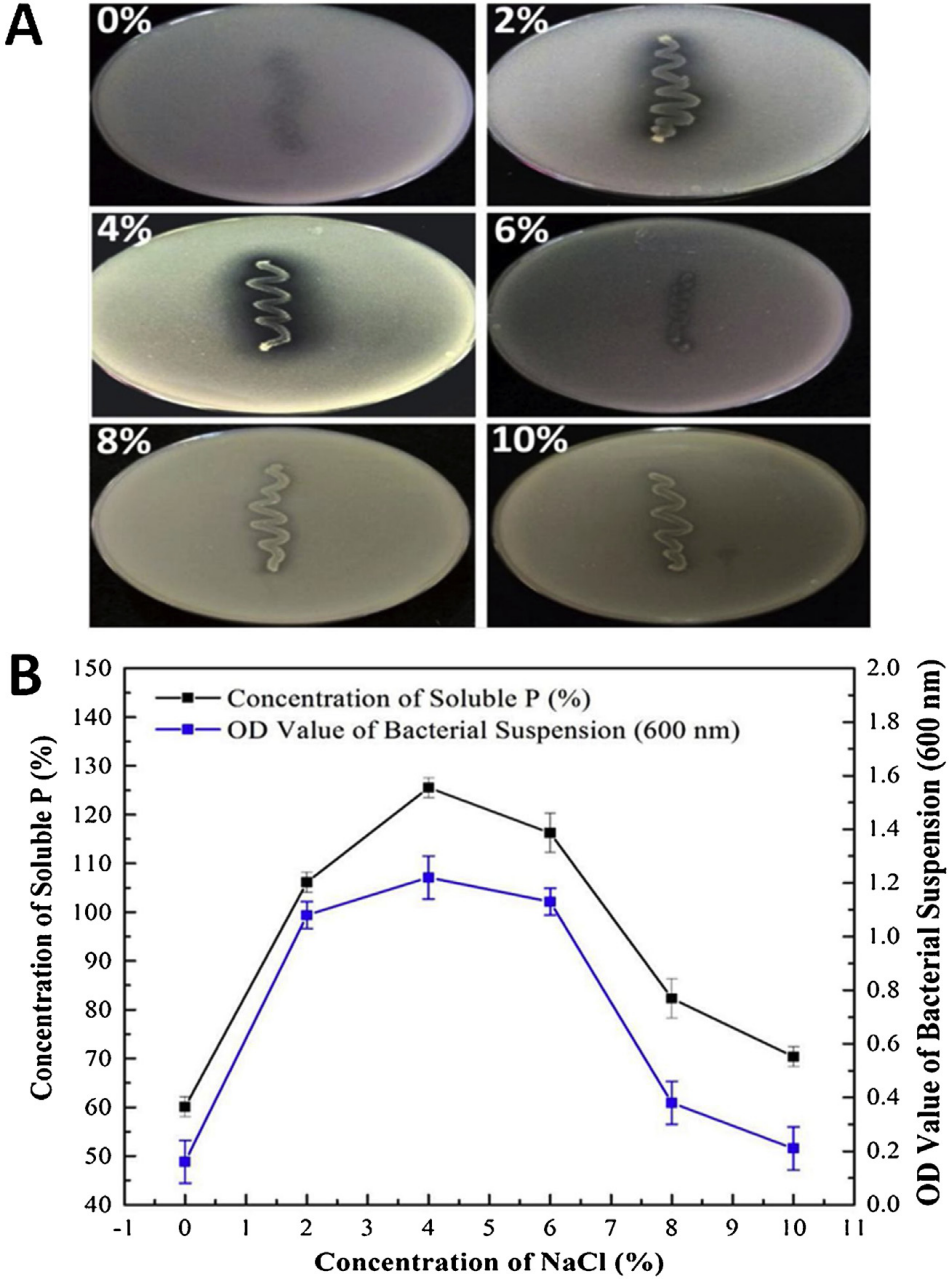
Effect of NaCl concentration on phosphate solubility. Bars represent standard deviation (SD). (A) Dissolved phosphate in Sperber Broth (SB) medium under various NaCl concentrations, (B) Jme3-20 strain cell growth relationship curve with dissolved phosphate.

In general, the majority of bacterial isolates were able to solubilize Ca_3_(PO_4_)_2_ supplemented in the Pikovskaya medium. However, solubilizing activities were different at 48–168 h of incubation for each isolate. Only seven isolates out of 35 showed the highest phosphate solubilizing activities within 48 h, while the remaining (28) showed either growth or solubilizing activities after 48 h. Clear zones formed around colonies indicated phosphate solubilization positive results due to organic acids or bacteria enzyme production (Fig. 7, Fig. 8).

### 3.8 Screening of ureolytic bacteria

**Fig. 9:**
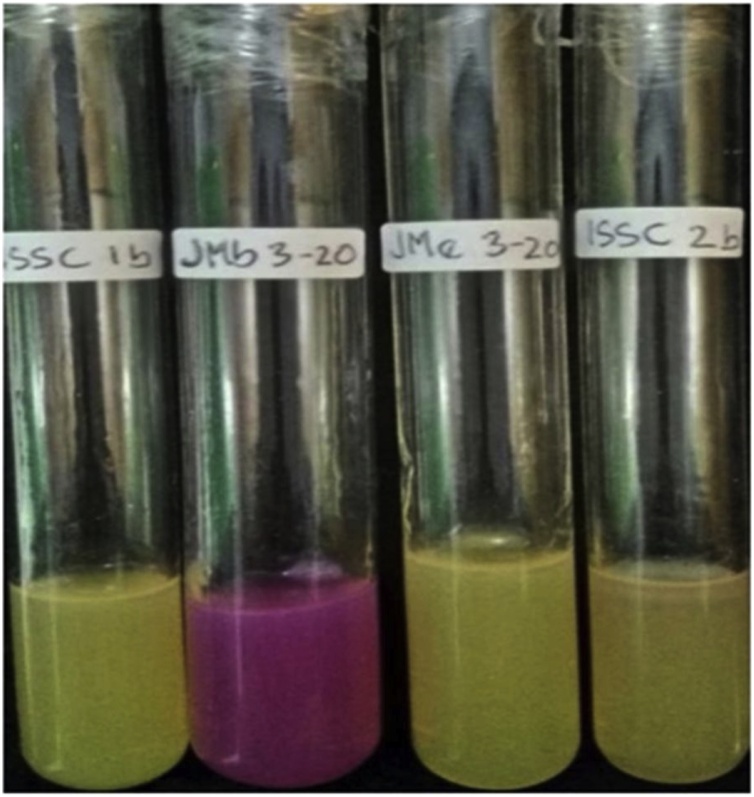
Jmb3-20 showing color change, from yellow to pink, indicating positive results regarding urea hydrolysis.

Four isolates were observed as urease-producing strains in this study (Table 3). The duration of urea hydrolysis was 72-h before showing positive results. Color change from yellow to pink indicated urea hydrolysis positive results. Urea was used as a nitrogen source in the medium (Fig. 9).

### 3.9 Effect of salinity on phosphate solubilization by bacterial strain

The effect of salt concentration on cell growth and phosphate solubilization was studied with supplementation of media with NaCl in a range of 0–10 % with an increment of 2 %. The highest cell growth and phosphate solubilization (125.51 mg/L) were observed at 4 % supplementation indicating 8A shows growth onto plates and 8B shows the relation of Jme3−20 strain with dissolved phosphate curve.

### 3.10 Optimization of protease production by Jme3−20

**Fig. 10:**
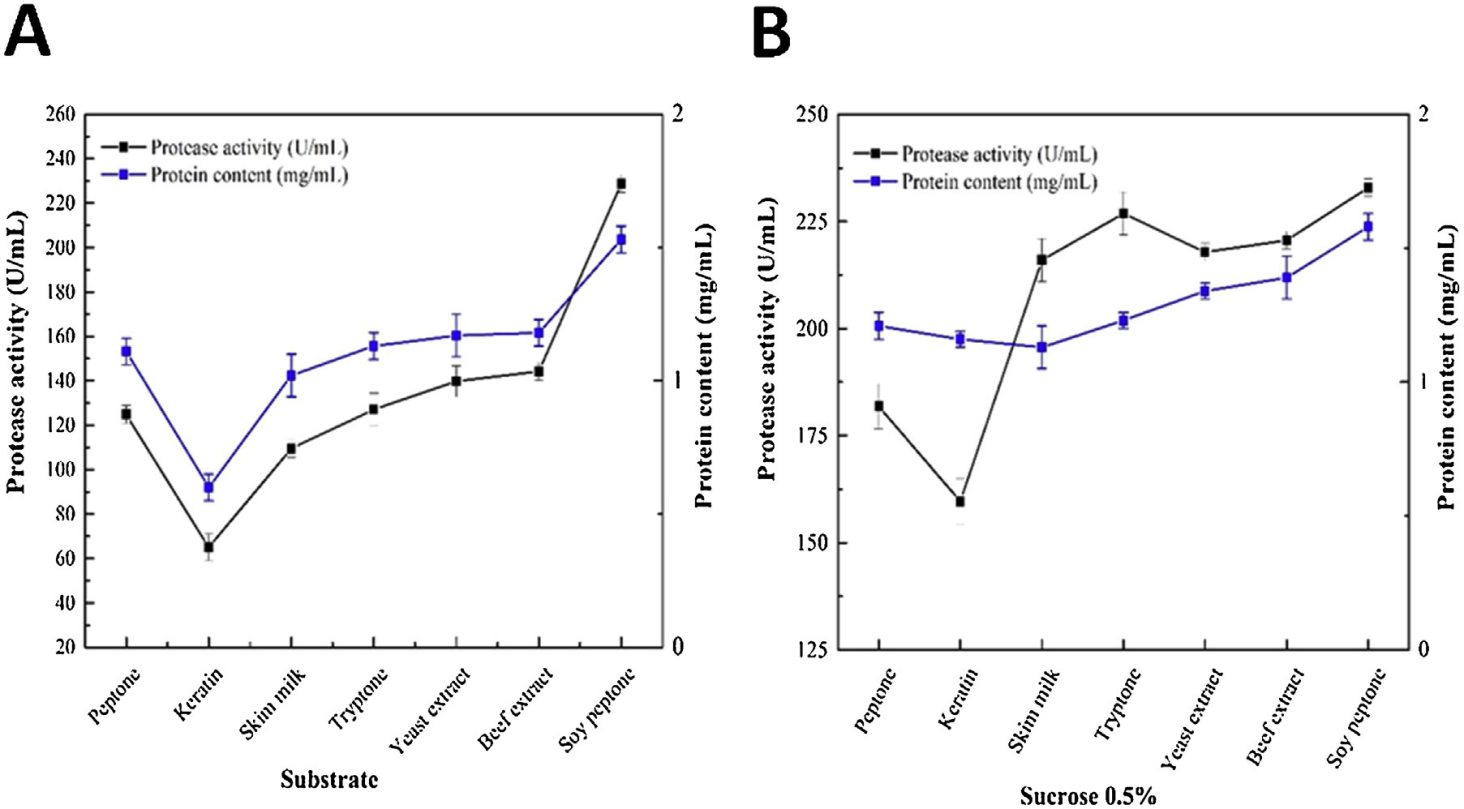
Influence of different substrate sources on Jme3-20 protease activity and protein content (A) without and (B) with sucrose 0.5 % after 24 h incubation.

**Fig. 11:**
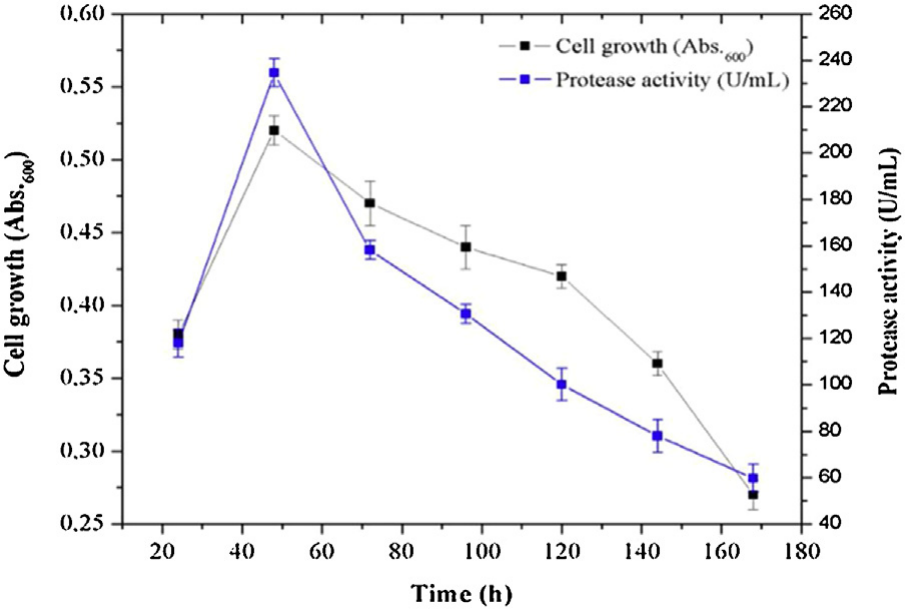
Correlation of Jme3-20 bacterial growth to protease activity, after supplementation with soy peptone. Cell growth and protease activity. Optimum cell growth and protease activity was observed at 48 h.

Optimization on protease production of the isolate Jme 3−20 utilized seven different substrates. The results showed that supplementation of soy peptone increased the specific protease activity and protein yield compared to other substrates. Soy peptone optimization produced the highest protease activity (228.81 U/mL) while the lowest activity obtained was by keratin supplementation (65.06 U/mL) (Fig. 10A). Meanwhile, supplementation with 0.5 % of sucrose into the medium resulted in higher activity (232.86 U/mL) than before given sucrose, in the addition of soy peptone (Fig. 10B). Furthermore, protease activity was observed at incubation time intervals. The optimum growth of isolate Jme3−20 was observed at 48 h, with elevated protease activity (Fig. 11). Sucrose 0.5 % (C-source) and soy peptone (N-source) were added into the growing medium.

### 3.11 Molecular identification of isolate Jme3−20

**Fig. 12:**
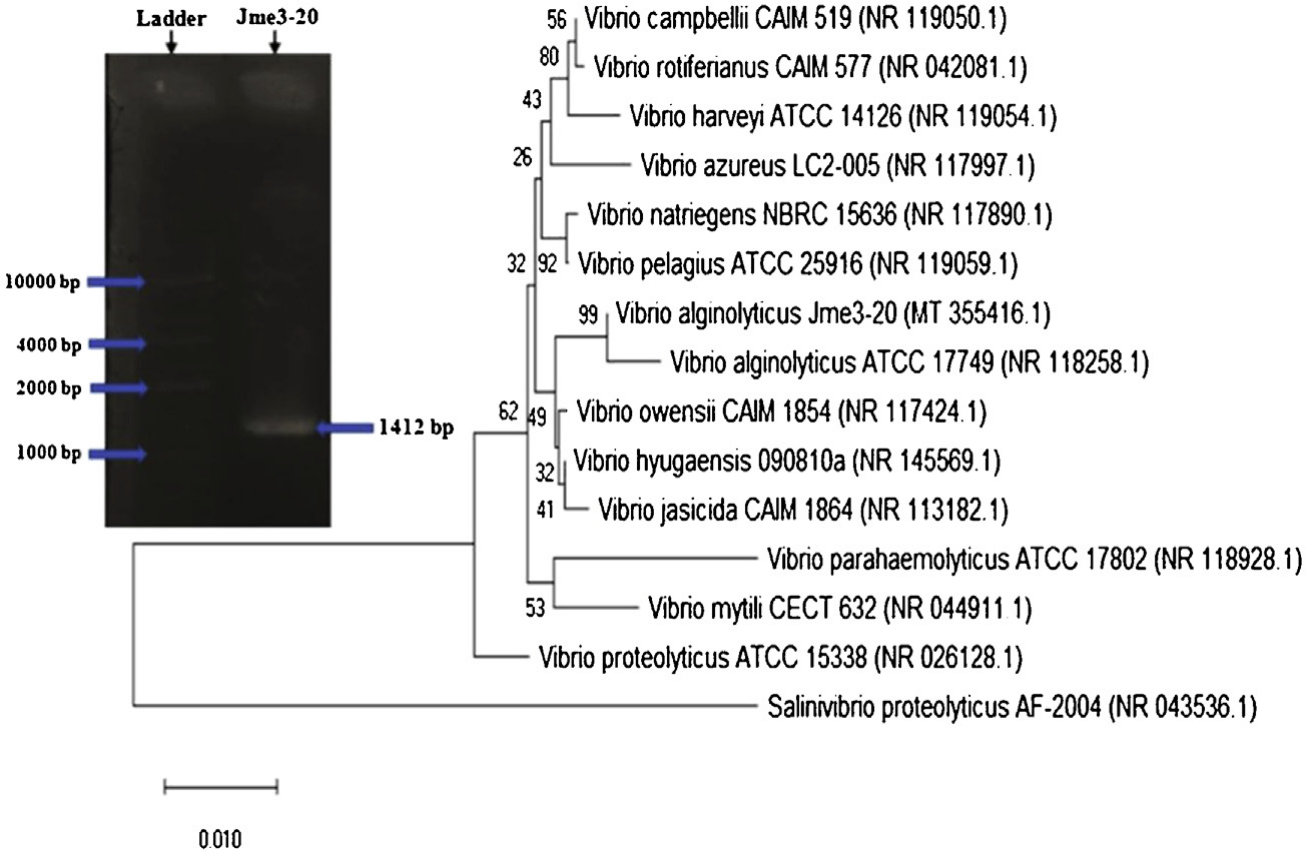
Phylogenetic tree based on the neighbour-joining method, using partial 16S rDNA gene sequences. Bootstrap values are expressed as percentages of 1000 replications; only values >50 % are shown. *Salinivibrio proteolyticus* was used as outgroup.

Isolate Jme3−20 was selected as the most potential strain among all tested strains associated with *Bruguiera cylindrica*. Molecular identification was performed by generating a phylogenetic tree to resemble its genetic distance or similarity, using databases retrieved from NCBI. The 16S rRNA gene amplicon, obtained by PCR using genomic DNA from the isolate Jme 3−20, was successfully purified after visibly determined to be a single DNA band (Fig. 12).

### 3.12 SDS-PAGE and zymography

**Fig. 13:**
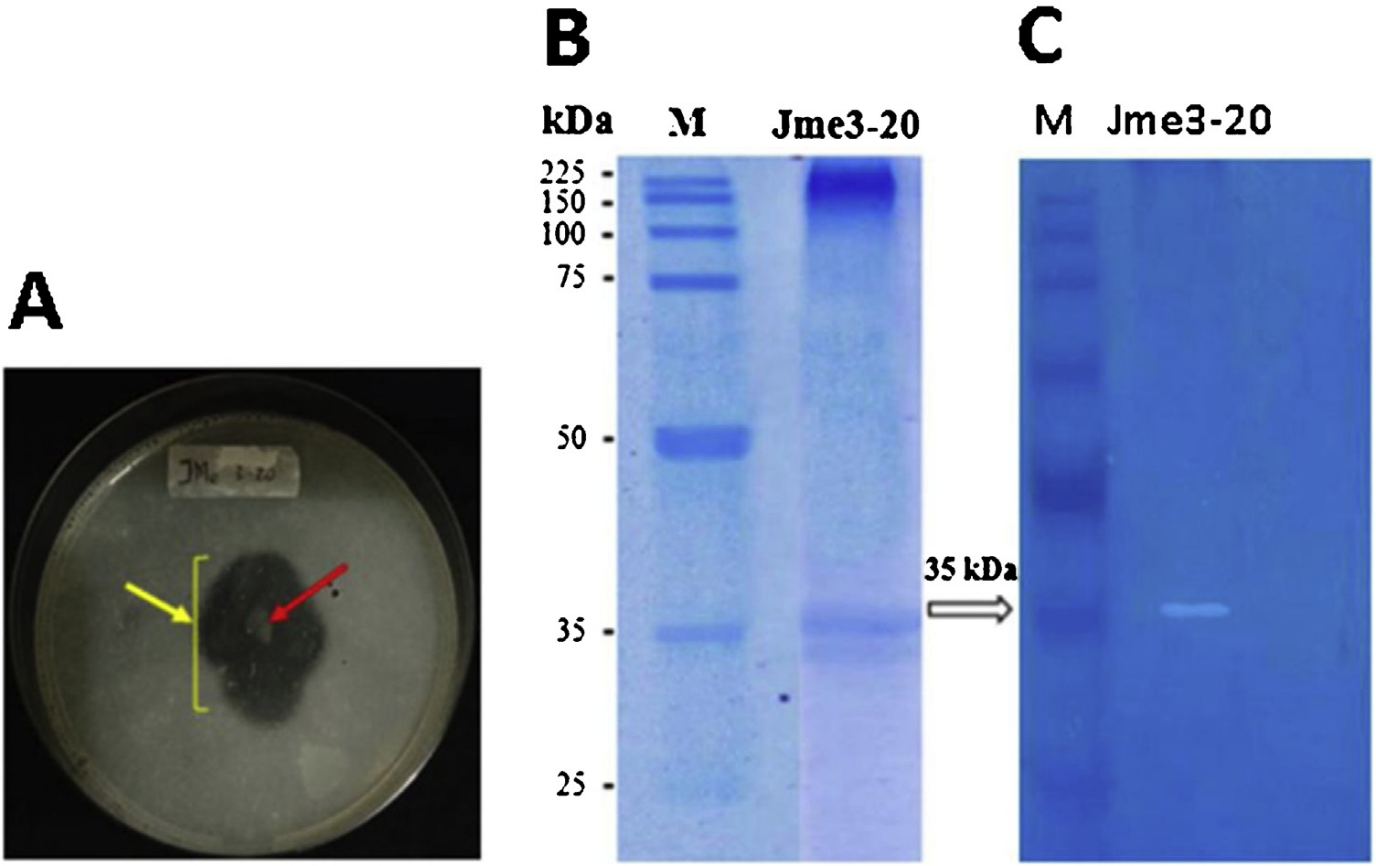
Detection of proteolytic degradation in casein agar plates and zymography. (A) Casein agar plates with Jme3-20 Vibrio isolate. Plates were stained with Coomassie brilliant blue reagent to identify the degradation of casein by proteases. The arrow (red; colony, yellow; clear zone) indicates the colony used for subsequent studies, (B) SDS-PAGE and (C) zymography of isolate Jme3-20 proteins. Lane M indicates proteins molecular weight marker. Samples were separated on a 10 % acrylamide gel.

The extracellular protease of *V. alginolyticus* Jme3−20 was purified into several fractions with the highest protein concentration being used to perform a protease zymography. SDS-PAGE and zymogram were aimed to confirm the presence of proteases and to determine its molecular weight. Results of SDS-PAGE separation are shown in Fig. 13 indicating the presence of two protein bands with a molecular weight range between 30–35 kDa. In the zymogram results (Fig. 13), it was observed that the proteolytic action of proteases toward casein substrates in a protein band of molecular weight of 35 kDa, therefore confirming the presence of the partial purified protease of *V. alginolyticus* Jme3−20 having a molecular weight of 35 kDa.

## 4 Discussion

This study aimed to isolate and characterize bacterial strains associated with the tree *Bruguiera cylindrica* from the mangrove forest of Pantai Gading Secanggang, Langkat regency, North Sumatra. To the best of our knowledge, this is the first report in evaluating multiple enzymes profiles, i.e. amylases, proteases, cellulases, chitinases, ureases, and phosphate solubilizing activities from associative bacteria isolated from this mangrove environment. Microorganisms around this habitat are interesting objects of study due to their physicochemical environmental conditions, which are distinctive from other ecosystems. Mangrove plants may alter microorganisms assemblage by developing specific adaptations to extreme environmental conditions, such as high temperatures, salinity fluctuations, tides, toxic pollutants, and serves as a biofilter of heavy metal pollutants [[32], [33], [34]]. In addition, the absorption of CO_2_ carbon in mangrove forests showed significant benefits in global carbon accumulation and reduces atmospheric CO_2_ [[35], [36], [37]]. Recently, halophilic microorganisms, especially bacteria, have attracted the attention of many researchers to find novel enzyme sources, since these could be relatively more stable and active than other enzymes derived from plants or animals [38,39]. Previous studies have reported many bacterial strains originated from mangrove environments, such as soils ([[40], [41], [42]]), endosphere [43], and rhizosphere [44]. However, the present study was focused on isolating bacteria from mangrove in different soils depth (10, 20, and 30 cm) and associated with some insects that inhabit the same area of the mangrove tree. Three isolates of mangrove bacteria (ISSC1b, ISSC2b, and Jme3−20 strains) were found to have the potential to produce four different enzymes (amylases, proteases, cellulases, and chitinases) and were able to dissolve insoluble phosphates. These strains were molecularly identified as members of the *Vibrio* genus. Specifically, the isolate Jme3−20 (Fig. 12) was further evaluated for optimized protease production. Molecular results showed that this strain presented high similarity to *Vibrio alginolyticus* (accession number MT355416). This bacterial strain was successfully recovered from mangrove soil at 20-cm depth. *Vibrio alginolyticus* is widely distributed in various marine environment [45], and some strains can cause diseases of marine animals [46]. Results of previous studies also succeeded in isolating members of the *Vibrio* genus from mangrove sediments ([47,48]). The strain, *Vibrio alginolyticus* Jme3−20 was characterized as an urease-negative bacterium with prominent amylolytic activities. The conversion of starch by α-amylase was faster than by β-amylase. This result was obtained through 24 h incubation and by measuring the breakage of the α-1,4-glycosidic link [49], therefore displayed a rapid starch hydrolysis. Interestingly, *Vibrio alginolyticus* Jme3−20 produced both α- and β-amylases, adding a new bacteria genus to the most studied amylolytic bacteria from genera *Bacillus, Streptomyces, Micrococcus, Arthrobacter, Escherichia, Pseudomonas, Proteus*, and *Serratia* [50,51]. In a previous report, Kanimozhi et al. [52] successfully recovered four strains of *Bacillus* sp from mangrove soils, with the ability to produce α-amylases. To date, the commercialization of α-amylases in industrial sectors is mostly derived from members of *Bacillus* [53], with a 50 % estimation of the total global market of α-amylases [54]. The demand for α-amylases (E.C.3.2.1.1) is increasing in the industrial sector due to its important role in starch hydrolysis into low molecular weight sugars [55]. The enzyme α-amylase has been widely used in various sectors to remove environmental pollutants, synthesize detergents, paper, alcohol, and in the textile and bread industry ([[55], [56], [57]]). Other enzymes produced by *Vibrio alginolyticus* Jme3−20 included proteases, cellulases, and chitinases. Qualitative screening of proteases produced by *Vibrio alginolyticus* Jme3−20 revealed that they were active after 21 h of incubation time, and the hydrolysis zone became clearer after 24 h of incubation. Cellulases were also produced by *Vibrio alginolyticus* Jme3−20, with a relatively longer initiation time of 96 h incubation. Reports on other *Vibrio* species capable of producing both proteases and cellulases have been done using *V. fluvialis* and *V. xiamenensis* [58,59]. Meanwhile, *Vibrio alginolyticus* NBRC which was successfully isolated from horseshoe crab, jellyfish and marine water produced only proteases [60]. Thus, the present study adds information about *Vibrio alginolyticus*, which has not been previously reported to produce proteases and cellulases. This may also indicate that the mangrove environment has a high diversity of microbes, although the exploration and sampling efforts may be limited to spatio-temporal patterns from each region. The strain *Vibrio alginolyticus* Jme3−20 was also found to have chitinase activity. This strain was the only isolate to produce chitinases after 6 days of incubation. Other members of *Vibrio*, with recorded chitinase activity have been reported, such as *V. harveyi, V. cholerae*, and *V. proteolyticus* [[61], [62], [63]]. Our results on *V. alginolyticus* Jme3−20 may be considered as the most recent report for chitinase-producing strain from the *Vibrio* genus. Another feature studied in all bacterial isolates was their ability to solubilize tricalcium phosphate (Ca_3_(PO_4_)_2_). Again, *Vibrio alginolyticus* Jme3−20 showed a faster phosphate solubilizing activity than other isolates, which took only 48 h of incubation. Reports of other phosphate solubilizing bacteria from the mangrove environment have identified members of the genera *Bacillus*, *Pseudomonas*, *Acinetobacter*, which were isolated from the rhizospheric area and mangrove sediments of Avicennia marina [64,65]. Other genera, i.e. *Serratia* and *Alcaligenes* were also reported as phosphate solubilizing bacteria [66]. Mangrove sediments play an important role as physical substrates and nutritional sources in mangrove ecosystems. Biochemical processes occurring in mangrove forests are especially from upper sediments, including oxidation processes and plant-soil interactions ([40,41]). Bacteria are involved in the process of P (phosphate) transformation in soil sediments and become an important part of the soil P cycle. In particular, soil bacteria are effective for releasing organic and inorganic P, from total soil P through dissolution and mineralization [67]. *Vibrio alginolyticus* Jme3−20 was isolated from sediments of *Bruguiera cylindrica* at 20 cm depth, which is still classified as the topsoil layer. This part of the layer is abundant in organic compounds, as a result of the decomposition of plant litter and dead animals. The availability of organic P components in a top layer of mangrove sedimen allows bacteria to use them as nutrients source. Currently, phosphate solubilizing bacteria can be applied in biofertilizer, nematicidal and fungicidal formulations [[68], [69], [70], [71], [72], [73], [74], [75]]. Most likely, *Vibrio alginolyticus* Jme3−20 has the potential to be applied in the future, considering that the utilization of phosphate solubilizing bacteria from the genus *Vibrio* is still limited. The present report comprises the isolation of *V. alginolyticus* Jme3−20 strain from mangrove ecosystem, a type of salt-affected ecosystem. Isolates from these habitats have a salt requirement and such ecosystems are also explored for salt-tolerant strains [76]. Phosphate solubilization is a potential activity of strains isolated from saline environments and thus preferred to be used in agriculture in saline-alkaline soils [77]. Phosphate solubilization involves a complex mechanism affected by various factors in which the salt concentration is one of the primary importances to halotolerant strains. Possessing phosphate solubilization characteristics are beneficial for different purposes. Quantitative assay of protease activity by *V. alginolyticus* Jme3−20 began with substrates optimization for enzyme production. Substrates used as N source were peptone, keratin, skim milk, tryptone, yeast extract, beef extract, and soy peptone. The results showed that the addition of soy peptone increased protease activity (Fig. 10A), accompanied by a high amount of protein production (Fig. 10A). The protease activity obtained was 228.81 U/mL and the total protein was 1.53 mg/mL within 24 h of incubation (Fig. 10A). When 0.5 % sucrose was added as a carbon source in the fermentation medium, protease activity slightly increased to 232.86 U/mL with a total protein of 1.58 mg/mL (Fig. 10B), compared to no sucrose addition (Fig. 10A). The optimization of protease activity was also observed at various incubation time intervals. The results indicated that 48 h of incubation was the best time to increase Jme3−20 protease activity. Information on protease activity is not limited to substrates testing. SDS-PAGE and zymogram tests can also be used. Interestingly, only a few reports have been published regarding the determination of protein molecular weights of isolates of the *Vibrio* genus. Even proteins from *Vibrio alginolyticus* have not been reported, which is the case of our strain Jme 3−20, molecularly identified as *Vibrio alginolyticus* (Fig. 12). SDS-PAGE results showed two partially purified proteins. Therefore, the zymogram analysis was performed to observe which one had protease activity on a specific protein substrate. Zymography exhibited only one protein, with a molecular weight of 35 kDa (Fig. 13C). Previous studies have reported similar results, but with the protease of different molecular weights such as a 36-kDa protease by *V. parahaemolyticus* [78], 110-kDa by *V. proteolyticus* [79], and 45 kDa in *Vibrio* sp. SJS2−3 [80]. Thus, this study became an early report of a 35 kDa Jme3−20 protease which actively hydrolyzed casein as substrate.

## 5 Conclusion

The present study revealed important insights into the bacterial diversity and activity in mangrove ecosystems, with the isolation and characterization of a great number of microorganisms associated with the plant *Bruguiera cylindrica*. These isolates displayed diverse adaptation features that could be useful for biotechnological applications such as enzymatic and phosphate solubilizing activities. Also, our results evidence the importance of microbial exploration in mangroves, since a better understanding of the ecosystem’s microbial diversity and functioning is of paramount importance in the pursuit of sustainable practices and preservation policies.

## Declaration of Competing Interest

The authors declare that there is no conflict of interest.

## CRediT authorship contribution statement

**Jendri Mamangkey:** Conceptualization, Methodology, Writing - original draft. **Dwi Suryanto:** Resources, Supervision, Writing - original draft. **Erman Munir:** Resources, Investigation. **Apon Zaenal Mustopa:** Methodology, Formal analysis, Project administration. **Mada Triandala Sibero:** Methodology, Formal analysis, Writing - review & editing. **Lucas William Mendes:** Writing - review & editing. **Adrian Hartanto:** Visualization. **Steven Taniwan:** Writing - review & editing. **Maria Julissa Ek-Ramos:** Data curation, Writing - review & editing. **Arman Harahap:** Writing - review & editing. **Amit Verma:** Validation, Writing - review & editing. **Edy Trihatmoko:** Formal analysis. **Wendry Setiyadi Putranto:** Resources, Investigation. **Lukas Pardosi:** Data curation. **La Ode Adi Parman Rudia:** Visualization.
